# SMT-738: a novel small-molecule inhibitor of bacterial lipoprotein transport targeting Enterobacteriaceae

**DOI:** 10.1128/aac.00695-23

**Published:** 2023-12-12

**Authors:** E. B. M. Breidenstein, N. Khan, T. Duffy, C. Coward, T. Avis, O. Abdulle, C.-M. Li, C. S. Mason

**Affiliations:** 1 Summit Therapeutics, The Works, Unity Campus, Cambridge, United Kingdom; 2 Summit Therapeutics, Menlo Park, California, USA; Bill & Melinda Gates Medical Research Institute, Cambridge, Massachusetts, USA

**Keywords:** SMT-738, bacterial lipoprotein transport, carbapenem-resistant *Enterobacteriaceae*

## Abstract

Carbapenem-resistant *Enterobacteriaceae* (CREs) are described by the Centers for Disease Control as an urgent threat, and there is a critical need for new therapeutic agents able to treat infections caused by these pathogens. Herein, we describe the microbiological profile, the mechanism f action, and the *in vitro* safety as well as the pharmacokinetic (PK)/PD profile of SMT-738, a small molecule belonging to a new chemical class. SMT-738 is active against Enterobacterales [including multi-drug-resistant *Escherichia coli* with 90% of isolates having a minimum inhibitory concentration (MIC_90_) of 1 µg/mL and *Klebsiella pneumoniae* 2 µg/mL] and inactive against a broad panel of Gram-negative and Gram-positive pathogens. SMT-738 displays rapid bactericidal activity (2–4 h) and has a low propensity for resistance development (less than ~10^−9^). Characterization of resistant mutants following exposure to SMT-738 identified mutations within the lipoprotein transport complex (LolCDE), a clinically unexploited and essential bacterial molecular target in Gram-negative bacteria. SMT-738 has a promising *in vitro* toxicology profile. Furthermore, PK studies demonstrated that when dosed intravenously, SMT-738 maintained exposure levels across infection sites (bloodstream/urinary tract/lung). Proof-of-concept studies across multiple murine *in vivo* infection models (bloodstream/pneumonia/urinary tract) demonstrated that SMT-738 significantly reduced the bacterial burden compared to baseline and vehicle control. SMT-738 represents a promising novel drug candidate being developed to address clinically challenging serious life-threatening infections caused by highly resistant *Enterobacteriaceae* including CRE.

## INTRODUCTION

With nearly three million infections and ~40,000 deaths annually in the US and 700,000 deaths worldwide, antimicrobial resistance continues to be a growing problem globally (1). Specifically, carbapenem-resistant *Enterobacteriaceae* (CREs) have been described by the Centers for Disease Control as an urgent threat. Treatment options for CRE, particularly those carrying New Delhi metallo-β-lactamases (NDM), are extremely limited (2). Global spread of acquired metallo-β-lactamases has the potential to escalate (3), and outbreaks have been observed in multiple countries. This further fuels the global concern associated with the spread of antimicrobial resistance and the ability to manage and control infection. *Enterobacteriaceae* are a family of Gram-negative bacteria responsible for causing infections across multiple indications, including urinary tract, bloodstream infections, and hospital-acquired pneumonias. Treatment typically consists of a combination of a β-lactam antibiotic and a β-lactamase inhibitor (BL/BLI) or the use of colistin, an antibiotic of last resort due to its renal toxicity (4, 5). Despite the high unmet need and the rapidly increasing incidence of resistance to existing antibiotics, novel new mechanism antibiotics addressing drug-resistant *Enterobacteriaceae* infections across the clinical development pipeline are very sparse (6). In fact, the majority are further variations of broad spectrum β-lactam antibiotics coupled with a β-lactamase inhibitor (BL/BLI combinations). These combinations, while effective in the short term, will eventually succumb to pre-existing resistance mechanisms. The continued investment into discovering and developing novel new mechanism antibiotics is of paramount importance in order to tackle the current unmet needs and create a sustainable pipeline of new innovative solutions for future generations. Here, we present data for SMT-738 (patent WO 2022/233886A1 compound I), a novel antibiotic that evolved from a hit identified from a high-throughput screening campaign. SMT-738 targets the bacterial lipoprotein transport complex LolCDE, which has the potential to treat serious healthcare-associated infections caused by *Enterobacteriaceae*. SMT-738 displays potent antimicrobial activity against clinically relevant strains of *Enterobacteriaceae* and exerts its bactericidal activity via a mechanism distinct from that of known classes of antibiotics. The microbiological selectivity of SMT-738 across a broad panel of bacteria can be rationalized by the degree of amino acid sequence homology of LolC and LolE.

LolCDE is a clinically unexploited target which is absent in human cells but is essential for bacterial lipoprotein transport in Gram-negative bacteria. This complex is essential for the transport of lipoproteins from the inner membrane to the outer membrane of Gram-negative bacteria (7, 8). In *E. coli,* the complex consists of a heterodimer of LolC and LolE (which are homologous) and two molecules of LolD. ATP hydrolysis by LolD causes a conformational shift in the complex resulting in lipoprotein translocation through the inner membrane to the chaperone LolA. The protein is then trafficked to LolB in the outer membrane into which it is incorporated. Lethality of Lol complex inhibition is primarily driven by mislocalization of the major outer membrane lipoprotein Lpp, which is transported by the complex, to the inner membrane. This results in the covalent attachment of Lpp to peptidoglycan, resulting in the disruption of cell-surface integrity (9). Deletion of *lpp* or prevention of the covalent attachment to peptidoglycan prevents lethality of Lol complex inhibition, and indeed, *lpp* mutations have been identified in isolates resistant to LolCDE inhibitors (10).

To help address the sparse pipeline of new antimicrobial agents, the objective of this study was to discover, characterize, and develop a novel molecule with potent antimicrobial activity against some of the most serious threat clinical isolates (NDM-carrying *Enterobacteriaceae*) and exemplify additional favorable microbiological, ADME, and proof-of-concept data.

## RESULTS

### SMT-738 has potent and highly selective antimicrobial activity *in vitro*

The antimicrobial activity of SMT-738 was profiled initially against in-house representative strains comprising *E. coli* NCTC 13441 [multi-drug resistant (MDR), clinical isolate, CTX-M-15 ESBL, uropathogenic *E. coli*] and *K. pneumoniae* NCTC 13438 (MDR, Class A KPC) and returned potent minimal inhibitory concentration (MIC) values of 0.39 µM (= 0.15 µg/mL) and 1.56 µM (= 0.61 µg/mL), respectively. Subsequently, SMT-738 was profiled alongside five established antibiotics, comprising both standard of care and recently approved/marketed agents against a panel of global clinically relevant MDR isolates of *E. coli* (*n* = 100) and *K. pneumoniae* (*n* = 100) (Table 1). The panel included MDR isolates obtained from infections of the urinary tract, the bloodstream, and the respiratory tract. These isolates covered ESBL-producing and CRE strains as well as isolates carrying resistance markers spanning additional antibiotic classes. The antimicrobial susceptibility MIC_90_ values are summarized in Table 1. SMT-738 showed potent *in vitro* activity across all the clinical isolates with MIC_90_ values of 1 µg/mL against *E. coli* and 2 µg/mL against *K. pneumoniae*. In contrast, amoxicillin/clavulanate, and the recently marketed combination meropenem/vaborbactam, demonstrated compromised activity against *K. pneumoniae* (MIC_90_ of >32 and >8 µg/mL, respectively). Colistin displayed an elevated MIC, particularly against *K. pneumoniae* (MIC_90_ = 16 µg/mL). Additionally, two respiratory isolates (carrying NDM-5 and TEM-OSBL) from Russia in 2019 both reported to be resistant to cefiderocol (MIC >64 µg/mL) were screened and shown to be susceptible to SMT-738 with MIC values of 0.25 and 0.5 µg/mL, respectively.

**TABLE 1:** MIC_50_, MIC_90_ panel as well as MIC range of 100 *E. coli* and 100 *K*. *pneumoniae* isolates (including both ESBL- and CRE-resistant strains)

Antibiotic	MIC_50_ (µg/mL)		MIC_90_ (µg/mL)		MIC range (µg/mL)	
	*E. coli*	*K. pneumoniae*	*E. coli*	*K. pneumoniae*	*E.coli*	*K. pneumoniae*
SMT-738	0.5	1	1	2	0.12–4	0.25–8
Amoxicillin/clavulanate	>32	>32	>32	>32	4–>32	1–>32
Trimethoprim/sulfamethoxazole	>32	>32	>32	>32	≤0.03–>32	0.06–>32
Ceftazidime/avibactam	≤0.12	0.5	1	2	≤0.12–>8	≤0.12–>8
Meropenem/vaborbactam	≤0.06	≤0.06	1	>8	≤0.06–>8	≤0.06–>8
Colistin	0.5	0.5	0.5	16	0.25–8	0.25–>32

These results demonstrated that SMT-738 is an antibiotic overcoming existing resistant determinants.

To further determine the utility of SMT-738 to inhibit bacterial growth of CRE threat isolates *in vitro*, a panel of contemporary clinical MDR NDM isolates (including NDM-1, NDM-4, and NDM-5) from India (2018) was tested. SMT-738 retained excellent potency against all isolates of *E. coli* (*n* = 15) and *K. pneumoniae* (*n* = 15). The MIC range against SMT-738 for *E. coli* and *K. pneumoniae* isolates was 0.25–1 and 0.5–1 µg/mL, respectively (Table 2; Table S1). In comparison, amoxicillin/clavulanate and the recently marketed counterparts, ceftazidime/avibactam and meropenem/vaborbactam, displayed an inferior profile in comparison to SMT-738 against the NDM panel of isolates as highlighted in Table 2.

**TABLE 2:** MIC range for selected NDM Indian *E. coli* and *K. pneumoniae* isolates

Antibiotic	Range (µg/mL)	
	*E. coli* (NDM)	*K. pneumoniae* (NDM)
SMT-738	0.25–1	0.5–1
Amoxicillin/clavulanate	>32	>32
Trimethoprim/sulfamethoxazole	0.006–>32	0.5–>32
Ceftazidime/avibactam	>32	>32
Meropenem/vaborbactam	16–>32	32–>32
Colistin	0.5–1	1–32

To establish the susceptibility across bacterial species, SMT-738 was profiled against a panel of 100 bacterial species which included commensals commonly found in the gut microbiome (Table S2). The results revealed that SMT-738 exhibited an activity spectrum predominantly displaying potent activity against members of the Enterobacterales order (MIC: 0.12–2 µg/mL), with the exceptions of *Edwardsiella tarda* (MIC: 32 µg/mL); *Hafnia alvei* (MIC: 32 µg/mL), *Morganella morganii* (MIC: >64 µg/mL), and *Proteus* sp. (MIC: 8–32 µg/mL). The Gram-negative and Gram-positive pathogens comprising anaerobic gut commensals such as *Actinomyces* sp., *Bacteroides* sp., *Clostridioides* sp., *Bifidobacterium* sp., *Peptostreptococcus* sp., and *Veillonella* sp. returned MIC values of >128 µg/mL, thus supporting the notion that SMT-738 is potentially a microbiome-sparing agent that avoids collateral damage to preserve the gut microbial diversity. In addition to the Enterobacterales order of bacteria, *Haemophilus influenzae* and *Haemophilus parainfluenzae* were the only exceptions where SMT-738 displayed potent activity with both returning an MIC of 1 µg/mL.

Additionally, susceptibility testing in the presence of human serum (20% and 50%) demonstrated that SMT-738 MICs were unchanged under these conditions (Table 3).

**TABLE 3:** MIC data in the presence of 20% and 50% human serum (HS) against *E. coli* and *K. pneumoniae*

	MIC (no HS) µg/mL	MIC (20% HS) µg/mL	MIC (50% HS) µg/mL
*K. pneumoniae* IHMA 1769429	2	1	2
*K. pneumoniae* IHMA 1770556	1	0.5	1
*E. coli* IHMA 2015405	0.5	0.25	0.25
*E. coli* IHMA 2051414	0.5	0.5	0.5

### SMT-738 displays a rapid bactericidal antimicrobial profile

Time-kill experiments were conducted to further investigate and characterize the microbiological profile of SMT-738. *K. pneumoniae* NCTC 13438 (MIC: 0.61 µg/mL) and *E. coli* NCTC 13441 (MIC: 0.15 µg/mL) were profiled at concentrations of 2× and 4× the MIC across a range of timepoints over a 24-h period.

**Fig 1:**
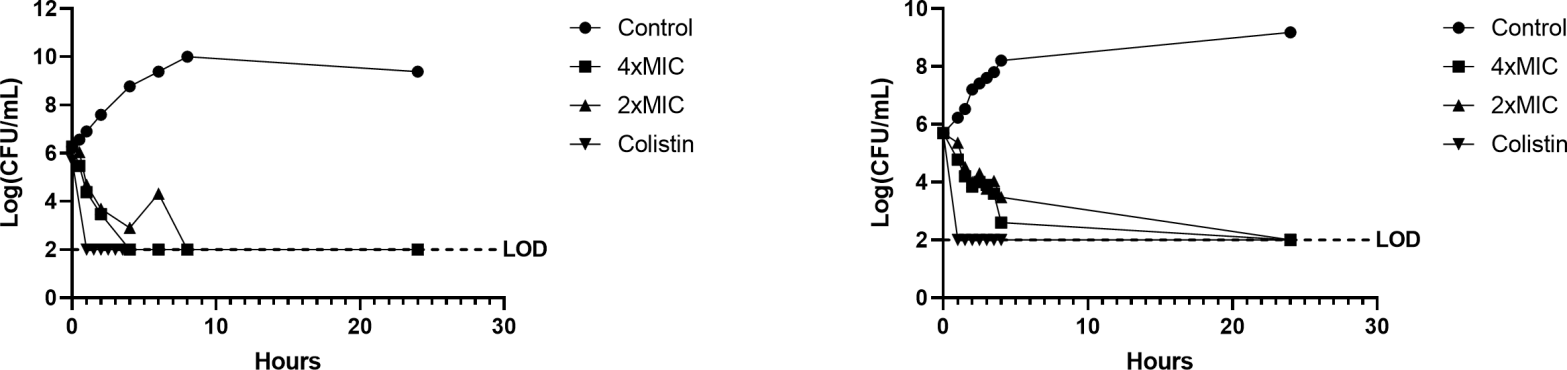
Time-kill curves of uropathogenic *E. coli* (UPEC) (left) and *K. pneumoniae* (right) in the presence of 2× and 4× MIC of SMT-738 and vehicle-only control over a 24-h period. The MIC of SMT-738 against UPEC used in this experiment was 0.39 µM (= 0.15 µg/mL) and against *K. pneumoniae* 1.56 µM (= 0.61 µg/mL). Colistin at 4× MIC served as the positive control for cidality. *E. coli* MIC was 0.16 µM (= 0.18 µg/mL) and for *K. pneumoniae* MIC was 1.56 µM (= 1.80 µg/mL). LOD, limit of detection.

SMT-738 at concentrations of 2× and 4× the MIC against *E. coli* displayed bactericidal activity at the 2-h timepoint, reducing viability by >3 log_10_ CFU/mL. This was maintained over the 24-h timepoint with no observed re-growth. In comparison, bactericidal activity was observed at the 4-h timepoint for *K. pneumoniae*. The bacterial burden remained below the limit of detection following 24-h exposure. A slight concentration-dependent effect was observed, with higher concentrations tending to result in a faster bactericidal response (Fig. 1).

### SMT-738 acts through a novel mechanism targeting lipoprotein transport

To elucidate the mechanism of action of SMT-738, isolates of *E. coli* and *K. pneumoniae* with reduced sensitivity were raised against SMT-738 at concentrations of 4× and 8× the MIC by single-step selection. Frequency of resistance (FoR) ~10^−9^ or lower was observed after 24 and 48 h (Table S3). Isolates were subjected to whole-genome sequencing to identify the specific mutations associated with reduced susceptibility to SMT-738. All isolates (10 for *E. coli* and 3 for *K. pneumoniae*) each had a single single-nucleotide variant (SNV) as compared to the wild type, which mapped to *lolC* or *lolE*. This suggested that the lipoprotein transport complex LolCDE, a clinically unexploited and essential bacterial molecular target, was the primary candidate for the drug’s molecular target. SNVs were identified in the periplasmic and transmembrane domains of LolE and in a transmembrane domain of LolC based on the annotations from Kaplan et al. (11) and Sharma et al. (7). All SNVs resulted in amino acid substitutions, and none were synonymous. Precisely, most mutated residues were located in transmembrane domain 2, clustering close to residues involved in the LolCDE complex’s interaction with lipoproteins. Alignments of LolC and LolE showing protein structural features and mutated residues are shown in Fig. S1. The identified mutants did show elevated MICs (Table 4). Two out of these mutations (LolC L256R and LolE L60P) are near residues previously identified as mutated in isolates resistant to other small-molecule LolCDE inhibitors. To further investigate genetic factors important for sensitivity to SMT-738, high-density transposon mutant screens exposing *E. coli* libraries to sub- and supra-MIC concentrations of SMT-738 were conducted (Fig. S2). These experiments revealed enrichment for mutants with the disruption of *lpp* or increased expression of *nlpE*. Deletion of *lpp* has previously been described as a resistance mechanism for small-molecule inhibitors of the LolCDE complex (10). Disruption of lipoprotein trafficking induces the Cpx envelope stress-response system via monitoring of trafficking by the NlpE(CutF) sensor lipoprotein (12). It is possible that increased expression of NlpE(CutF) could enhance this response, thus conferring a survival advantage in the presence of SMT-738. These observations support the hypothesis that SMT-738 acts by disruption of LolCDE complex activity.

**TABLE 4:** Locations and amino acid changes of mutated residues in isolates from mutation frequency experiments against SMT-738 are shown for both LolC and LolE in UPEC (*E. coli* NCTC 13441) and KPM (*K. pneumoniae* NCTC 13438) with the respective MIC fold change increase in brackets

LolC		LolE	
UPEC	KPM	UPEC	KPM
L256R (16×)	E198K (64×)	D264A (128×)	L60P (16×)
			M261R (>64×)

**Fig 2:**
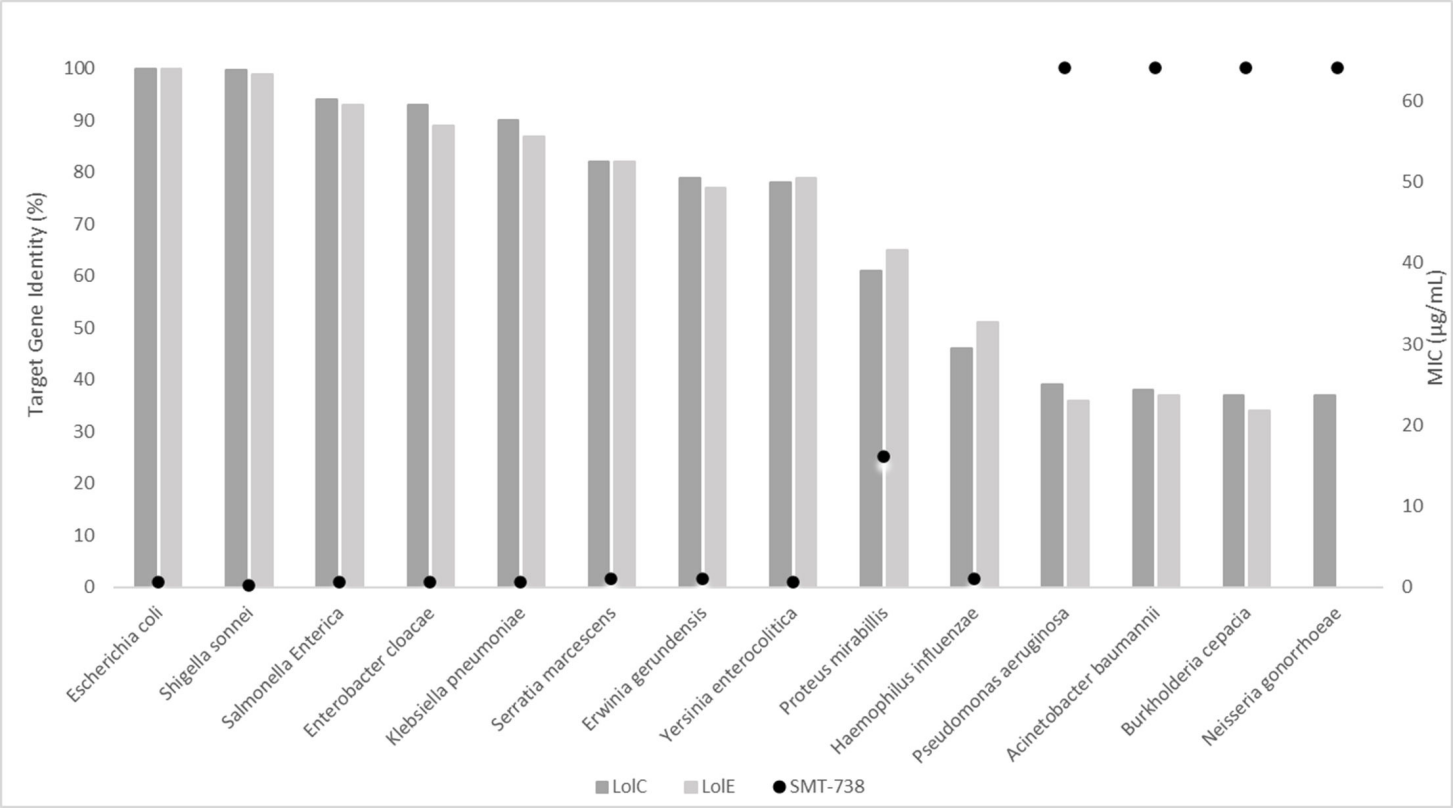
Relationship between antimicrobial activity and sequence identity for selected strains. Left *y*-axis, LolCE sequence identity; right *y*-axis, MIC (µg/mL).

There was a relationship between activity and LolC/E amino acid sequence conservation (Fig. 2). Sequence identity above 65% (across members of the *Enterobacteriaceae*) correlated with potent activity, whereas SMT-738 was inactive on species with identity below 50% (non-*Enterobacteriaceae*). *Haemophilus* was an exception to this trend, with SMT-738 showing activity despite overall sequence identity <50%. This could reflect a specific conserved binding site or protein structural features important for activity.

### SMT-738 has a favorable *in vitro* safety profile

To evaluate its promise as an antibiotic, SMT-738 was subjected to *in vitro* safety studies. First, the dose-dependent effect of SMT-738 on the viability of the mammalian cell lines HepG2 and HEK293 was investigated. The cytotoxicity IC_50_s for both cell lines were determined to be >200 µM (= 78.74 µg/mL). Thioridazine served as the positive control in the assay with an IC_50_ of 22.10 µM (= 8.19 µg/mL). Subsequently, SMT-738 was assessed against established mammalian cell assays to determine the potential of SMT-738 to cause genotoxicity, mitotoxicity, and nephrotoxicity. No toxicity was observed at the top concentration of 500 µM (= 196.85 µg/mL), and the cell health parameters were normal.

### SMT-738 distributes to key infection sites *in vivo*

**Fig 3:**
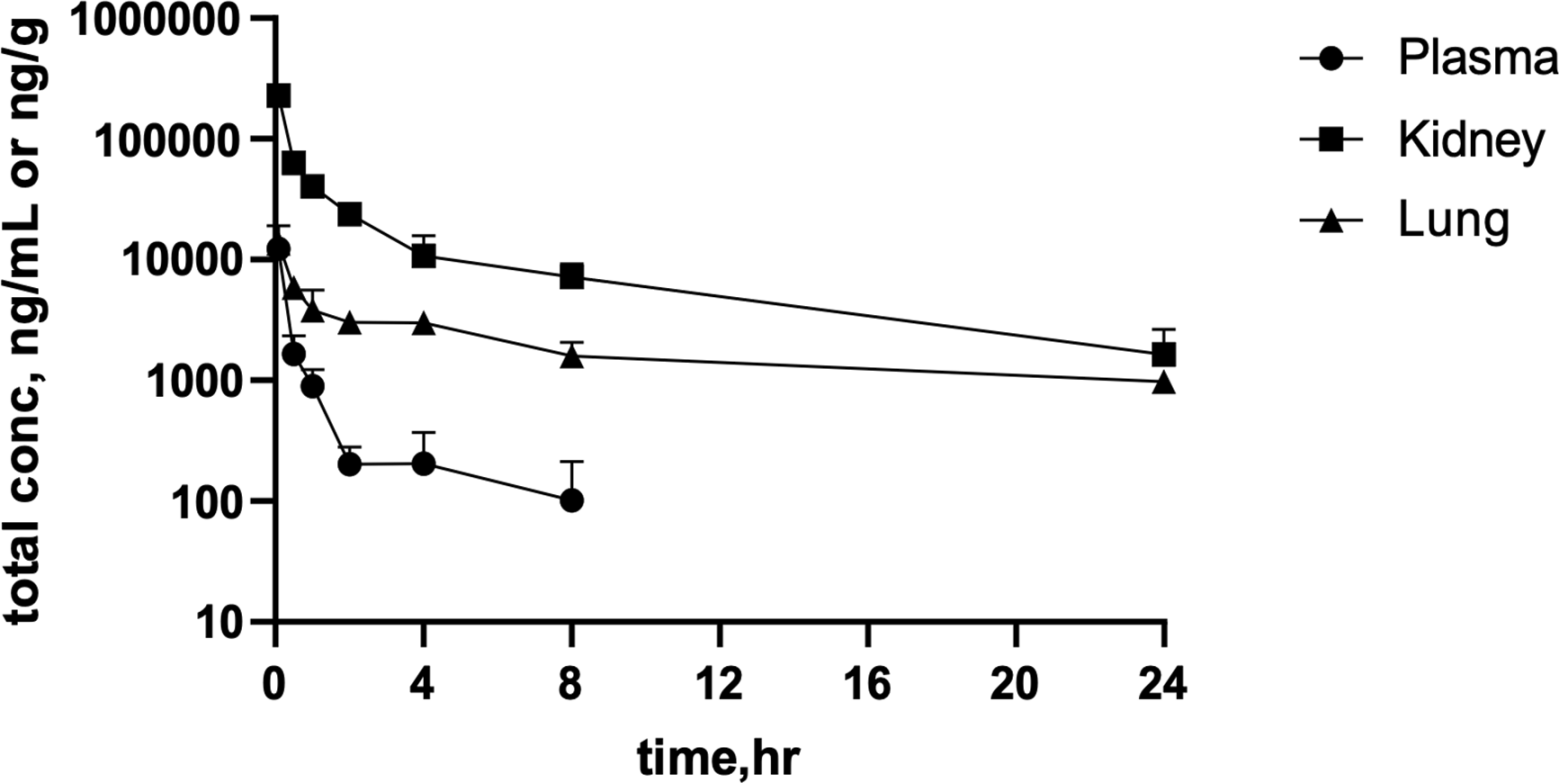
SMT-738 concentration-time profiles in plasma, kidney, and lung after IV bolus administration.

To ascertain the potential of SMT-738 to treat infections caused by *Enterobacteriaceae*, the pharmacokinetic (PK) profile of SMT-738 was evaluated. The drug concentration levels across the urinary tract, the bloodstream, and the lungs were determined in male CD-1 mice (Fig. 3; Fig. S3). In summary, the PK profile of SMT-738 demonstrated distribution to the target infection sites following a 20-mg/kg bolus IV administration (*C*_0_ = 18,386 ng/mL, *V*_ss_ = 4.3 L/kg, and CL = 59 mL/min/kg in plasma). SMT-738 displayed high tissue partitioning at both kidney and lung, with *K*_*p*,kidney_ and *K*_*p*,lung_ [based on the area under the curve (AUC) ratio] of 44.8 and 8.1, respectively. Most importantly, over a 24-h period, approximately 29.5% of SMT-738 was eliminated unchanged into the urine in mice. Furthermore, protein binding against mouse, rat, dog, and human has established and revealed 66.9%, 64.4%, 50.6%, and 62.7% of binding, respectively.

These findings support the potential of SMT-738 to treat infections caused by *Enterobacteriaceae*.

### SMT-738 demonstrates robust efficacy in relevant *in vivo* infection models

**Fig 4:**
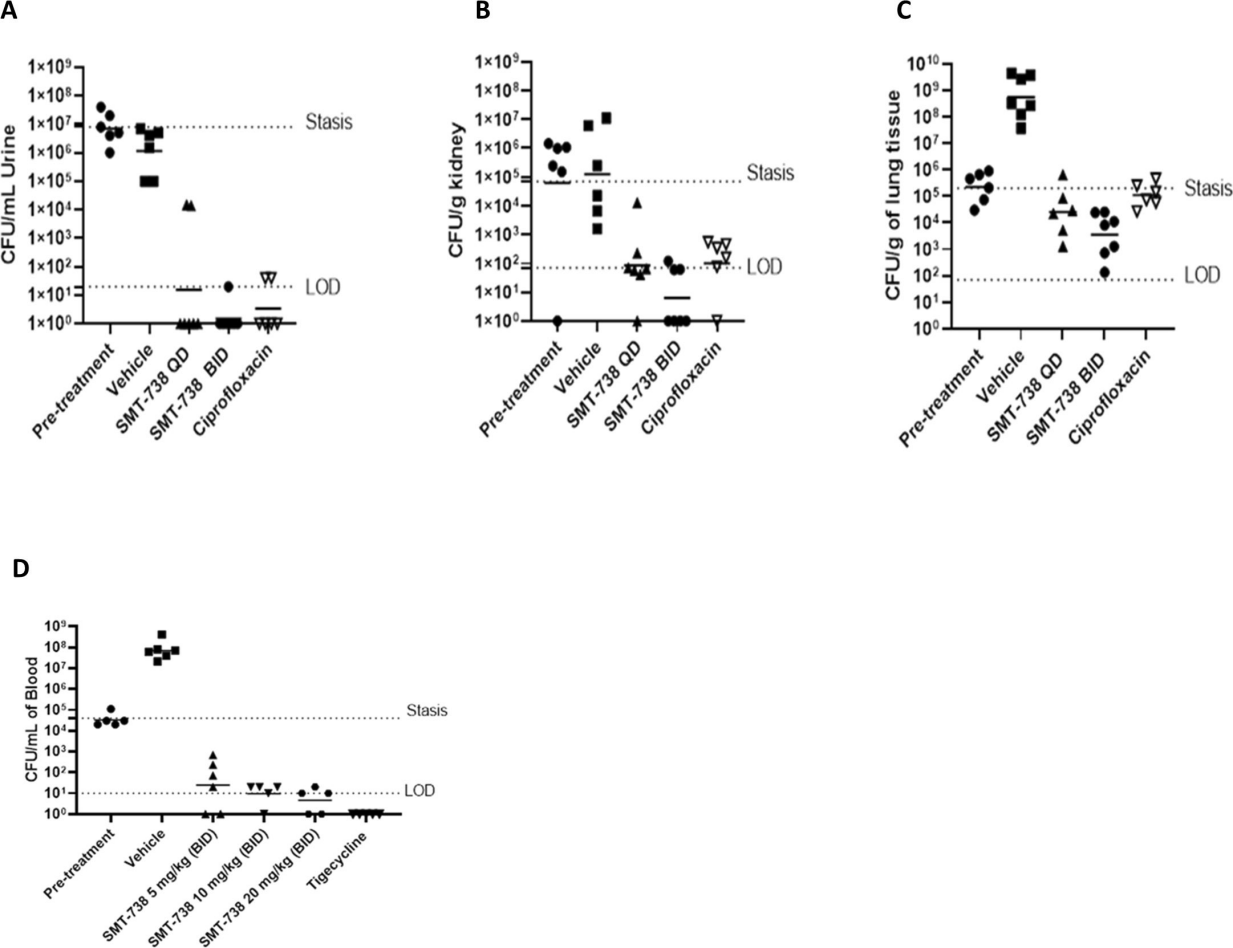
Proof of concept in murine urinary tract infection model (A and B), lung infection model (C), and bloodstream (D) given via IV infusion [20 mg/kg per dose (QD/BID)]. 20% Hydroxypropyl-β-cyclodextrin or phosphate buffer pH 6.0 was used as formulation vehicle for SMT-738. Solubility was >20 mg/mL. Reference compound ciprofloxacin was given IV bolus 10 mg/kg per dose BID and tigecycline IV bolus 40 mg/kg per dose BID. All treatment groups of SMT-738 showed statistically significant decreases from pre-treatment and vehicle control with *P*-values of <0.05.

Given both the striking *in vitro* microbiological profile and the favorable *in vivo* PK properties, SMT-738 was evaluated across three relevant *in vivo* infection models comprising urinary tract (*E. coli*), bloodstream (*E. coli*), and lung (*K. pneumoniae*). Initially, a foundation IV bolus efficacy study was conducted, which was followed up with further PK studies that identified IV infusion over 60 min as the optimal route of administration, reflecting what is anticipated for administering the dose/dosing regimen in the clinical setting. For the murine UTI study [female CH_3_/HeN mice infected with *E. coli* UTI89 (SMT-738 MIC: 0.5 µg/mL), readout 96 h], a daily QD or BID dose of 20 mg/kg administered by 60-min IV infusion over 3 days resulted in a robust clearance of the bacterial burden achieving levels below the limit of detection (5–6 log_10_ reduction compared to pre-treatment) in the urine and kidney (Fig. 4). In the murine bloodstream infection model [male CD-1 mice infected with *E. coli* BAA 2469 (NDM-1 positive, SMT-738 MIC: 0.5 µg/mL), readout 9 h], a dose-dependent response in the blood was established following BID 60-min IV infusion of 5, 10, and 20 mg/kg. The results indicated that 10 mg/kg given BID was almost as efficacious as the 20 mg/kg BID dose (reduction of 4 log_10_ compared to pre-treatment), both achieving levels below the limit of detection (Fig. 4). In comparison, SMT-738 in the murine lung infection model [male CD-1 mice infected with *K. pneumoniae* ATCC 43816 (SMT-738 MIC: 0.5 µg/mL)] administered by 60-min IV infusion either QD or BID at 20 mg/kg per dose resulted in a reduction of the bacterial burden of 1–2 log_10_ below pre-treatment stasis level at 24 h post-infection (Fig. 4). The data from the three respective studies strongly support the potential of SMT-738 to treat infections caused by *Enterobacteriaceae*.

## DISCUSSION

Ever since their discovery in the early 20th century, antibiotics have been the principal tools for treating bacterial infections. However, over recent decades, antibiotic resistance has risen alarmingly (13, 14), and the spread of multi-drug resistance has rendered a growing list of antibiotics ineffective in the treatment of antibiotic-resistant bacterial infections. This is coupled with, and compounded by, an incredibly sparse pipeline of new antimicrobials that are either in development or have recently gained approval. The rise of resistance and spread of acquired resistance markers [e.g., metallo-β-lactamases (3)] lead to the fact that antimicrobial resistance is considered to be a global healthcare concern. There is a real and urgent need for new antibiotics that exert their impact through new, targeted mechanisms to overcome established resistance mechanisms. To this end, the proteins involved in Gram-negative bacterial outer membrane biogenesis are attractive potential targets due to their essentiality. The LolCDE complex is composed of one copy each of LolC and LolE and two copies of LolD (15) with amino acid sequence identity between LolC and LolE of 26% (16, 17). In the work presented here, we describe the microbiological profile of a novel compound (SMT-738) with antibacterial activity against *Enterobacteriaceae* through targeting the lipoprotein transport complex (LolCDE). Inhibition of the Lol complex results in cell death due to the disruption of cell-surface integrity as a consequence of inappropriate crosslinking of the outer membrane protein Lpp to the inner membrane (9, 10). Using induced expression of an Lpp variant that crosslinks to the inner membrane, Yakushi et al. showed that mislocalization of this protein was rapidly bactericidal, at least in exponentially growing *E. coli* (9). Consistent with this, SMT-738 displayed rapid (within 4 h) bactericidal kinetics in time-kill experiments, similar to those reported (as data not shown) for a Lol complex small-molecule inhibitor (18).

The activity spectrum of SMT-738 is achieved by targeting this novel mechanism of action. Sequence identity appears to suggest that the antimicrobial activity is dictated by conservation of amino acid sequence across the target proteins (LolC/E), resulting in a profile that is predominantly selective for Enterobacterales.

SMT-738 (19) represents a novel, first-in-class, new mechanism of action, small-molecule antibiotic that overcomes current clinically relevant resistance mechanisms including all Ambler β-lactamase classes. Through susceptibility testing of clinically relevant and highly resistant isolates, we have demonstrated that SMT-738 retains potent activity against even the most challenging of *E. coli* and *K. pneumoniae* global isolates. We compared the activity of SMT-738 against established agents and demonstrated that SMT-738 shows increased potency compared to these marketed agents. Of importance, SMT-738 retained activity against the most difficult-to-treat isolates (carrying NDM) where marketed agents lack potency. The low mutation frequency (~10^−9^ or lower) and activity against clinically relevant strains for SMT-738 contrast with previously described LolCDE inhibitors that lacked potency against clinically relevant strains (10, 18). Nickerson et al. hypothesized that the high mutation frequency observed in their findings was inherently linked to inhibition of the LolCDE complex. Our findings clearly establish that SMT-738 exhibits a low mutation frequency and provide evidence that the LolCDE complex is a viable target with potential clinical application. Our mechanistic target assignment to date is only based on resistance selection data and currently lacks supporting biochemical or target interaction evidence.

Furthermore, SMT-738 is eliminated unchanged in the urine, distributes to the lung, and maintains sufficient levels in the bloodstream to suggest that relevant infection sites (urinary tract, bloodstream, and lower respiratory system) can be targeted with this one compound. Indeed, SMT-738 gave a robust efficacious response resulting in a substantial reduction in bacterial burden across murine infection models of UTI, bloodstream, and lung. SMT-738 has the potential to become an effective treatment option for infections caused by *Enterobacteriaceae*.

## MATERIALS AND METHODS

### Minimum inhibitory concentration

MIC tests were performed by broth microdilution in-line with CLSI susceptibility testing standards (20, 21) either in-house or at IHMA (Europe and USA) or JMI Laboratories using cation-adjusted Mueller Hinton Broth.

### Time-kill experiment

An overnight culture of the test strain was diluted to a viable cell density of ~10^6^ CFU/mL and dispensed into 96-well plates containing dilutions of SMT-738 to yield the specified multiple of the MIC. Plates were sealed with a breathable seal and incubated under shaking conditions in a humid incubator at 37°C. At specific timepoints (*t* = 0, 1, 2, 4, 6, 8, and 24 h), samples were taken and plated onto MH agar plates, and colonies enumerated after overnight growth at 37°C. Viable cell numbers as log_10_ (CFU/mL) were then plotted against time. Where no colonies were detected, the limit of detection was 100 CFU/mL. Compound activity was scored as bactericidal if the viable cell count decreased by at least 3 log_10_ from the initial count.

### Frequency of resistance determination and whole-genome sequencing

The FoR of bacterial strains to test compounds was determined by plating aliquots of overnight cultures onto MH-agar plates containing dilutions of the test compound at various multiples of reference MIC as previously determined. Total viable counts of the overnight cultures were determined by spotting 10–20 µL of decimal dilutions in 1× phosphate-buffered saline (PBS) onto non-selective agar. Assay plates were incubated for up to 48 h with colonies being enumerated every 24 h. FoR was determined by dividing the number of colonies observed by the total number of viable cells plated. Representative colonies were then picked and streaked onto selective agar to confirm resistance and obtain resistant isolates. Isolates growing on selective agar were then used to initiate overnight cultures, bacteria pelleted by centrifugation, supernatant removed, and pellets stored at −80°C for subsequent DNA extraction and whole-genome sequencing. Genomic DNA was extracted using the DNeasy Blood and Tissue Kit (Qiagen), quantified using the Quant-iT TM PicoGreen TM dsDNA Assay Kit (Thermo Fisher Scientific), and normalized to 0.2 ng/µL in nuclease-free water. Library preparation and barcode addition were with the Nextera XT DNA Library Prep Kit (Illumina). KAPA Pure beads (Roche) were used for DNA purification. Samples were pooled to achieve 100× coverage of each sample and run on an Illumina Nextseq 500/550 Mid Output Kit v2.5 (150 cycles) (Illumina).

### *In vitro* safety: cytotoxicity, genotoxicity, mitotoxicity, and nephrotoxicity

#### Cytotoxicity

HepG2 (ATCC, HB-8065) and HEK293 (ATCC, CRL-1573) were sourced from ATCC. For the cytotoxicity assay, cells were seeded onto 96-well plates, which were then incubated for 24 h in a 5% CO_2_ incubator at 37°C. On the day of the assay, 10-point, twofold serial dilutions of test compound were prepared in assay media (MEM + 10% FBS) in separate 96-well plates. Assay plates were removed from the incubator, and the media from all of the wells were removed, taking care not to disturb the cell layer. Compound-containing assay media or control media were added to the relevant wells. Assay plates were returned to the 5% CO_2_ incubator at 37°C for 24 h. CellTiter-Glo reagent (Promega, G7570) was used to determine the viability of the cells.

#### Genotoxicity

The standard GreenScreen genotoxcity assay was performed by Cyprotex, UK. The assay uses two strains of human lymphoblastoid TK6 cells, the test strain (GenM-T01), and the non-fluorescent control strain (GenM-C01). Incorporated in the test strain is a patented green fluorescent protein (GFP) reporter system that exploits the proper regulation of the *GADD45a* gene, which mediates the adaptive response to genotoxic stress. Exposure to a genotoxic compound increases expression of GFP and, hence, the induction of cellular fluorescence in the test strain.

#### Nephrotoxicity

The standard nephrotoxicity assay was performed by Cyprotex, UK. Renal proximal tubule epithelial cells were exposed to a range of concentrations of compound, loaded with relevant dye/antibody for each cell health marker and scanned using a fluorescent cellular imager (ArrayScan, Thermo Scientific Cellomics). Cell health parameters that were analyzed: cell count, nuclear size, DNA structure, mitochondrial mass, mitochondrial membrane potential, phospholipidosis, glutathione content, and cellular ATP.

#### Mitochondrial toxicity

The Seahorse mitochondrial functional assay was performed by Cyprotex, UK. HepG2 cells were dosed with test compound, and in real time, the extracellular oxygen levels and pH were measured using the Xfe96 flux analyzer (Seahorse Biosciences). Simultaneously, oxygen consumption rate and extracellular acidification rate were measured to determine its effect on oxidative phosphorylation.

### Pharmacokinetics

Male CD-1 mice (from Charles River, Margate UK) were administered 20 mg/kg of SMT-738 via IV bolus route. At selected timepoints, blood, kidney tissue, lung tissue, and urine were collected and progressed for further analysis. The PK parameters included initial concentration (*C*_0_) via extrapolation, clearance (CL), volume of distribution (*V*_ss_), and AUC_0-*t*_ in plasma were determined using non-compartmental analysis. Tissue partitioning values were determined with ratio of AUC_0-*t*,kidney_/AUC_0-*t*,plasma_ for kidney (*K*_*p*,kidney_) and ratio of AUC_0-*t*,lung_/AUC_0-*t*,plasma_ for lung (*K*_*p*,lung_). The mean recovery (%) of SMT-738 unchanged in urine was determined by amount in urine compared to the intravenously administered dose. The study was conducted by Evotec Ltd (UK).

### Murine *in vivo* proof of concept: UTI, bloodstream, and lung infection

#### UTI infection

Female JVC (jugular venous catheter)-cannulated C_3_H/HeN mice were placed on drinking water which was supplemented with 5% glucose solution prior to infection. Mice were infected transurethrally with *E. coli* UTI89 following published methods. SMT-738 was intravenously infused over 60 min once (QD) or twice (BID, 4 h after the end of first infusion) per day for 3 days starting 24 h post-inoculation. The mice from pre-treatment group were euthanized (with an overdose of pentobarbitone) at 24 h, the remaining mice were euthanized at 96 h post-infection. The kidney, bladder, and urine were collected and quantitatively cultured by plating serial dilutions on appropriate media; CFU were counted after overnight incubation at 37°C. A Kruskal-Wallis statistical test was performed.

#### Bloodstream infection

Male JVC-cannulated CD-1 mice were infected intraperitoneally with *E. coli* BAA2469. At *t* = 1 h and *t* = 6 h, SMT-738 was intravenously infused over 60 min (BID). The mice from the pre-treatment group were euthanized at 1 h post-infection. All remaining mice were euthanized at 9 h post-infection, and blood samples were collected via cardiac puncture. Blood samples were serially diluted in PBS and plated onto cystine-lactose-electrolyte-deficient (CLED) agar. The viable bacterial burden for each sample was determined following overnight incubation at 37°C. A Kruskal-Wallis statistical test was performed.

#### Lung infection

Male JVC-cannulated CD-1 mice were rendered neutropenic using cyclophosphamide on days −4 and −1 (150 and 100 mg/kg, respectively). Mice were infected intranasally with *K. pneumoniae* ATCC 43816, and SMT-738 treatment was initiated at QD, 2 h post-infection; BID, 2 and 5 h post-infection. The pre-treatment control animals were euthanized at 2 h post-infection. All other study animals were euthanized at 24 h post-infection. Lungs were dissected and homogenized and quantitatively cultured, serially diluted, and plated on CLED agar, and CFU were quantified following overnight incubation at 30°C. A Kruskal-Wallis statistical test was performed.

All animal studies were conducted by Evotec Ltd under UK Home Office License with local ethical committee clearance.

## SUPPLEMENTAL MATERIAL

Supplementary Fig. S1 aac.00695-23-s0001.ppt Clustal Omega alignment of LolC (A) and LolE (B) showing structural features and mutated residues.

Supplementary Fig. S2 aac.00695-23-s0002.ppt Artemis plot showing the location of mini-transposon insertions around lpp (A) and nlpE(cutF) (B) in *E. coli* NCTC 13441 libraries treated with SMT-738.

Supplementary Fig. S3 aac.00695-23-s0003.ppt SMT-738 concentration-time profiles in plasma after IV infusion administration.

Supplementary Table S1 aac.00695-23-s0004.pdf Isolates used in Table 2.

Supplementary Table S2 aac.00695-23-s0005.pdf Minimum inhibitory concentration (MIC μg/mL) against 100 clinical bacterial species.

Supplementary Table S3 aac.00695-23-s0006.pdf Frequency of resistance (or limit of detection) for SMT-738 at the indicated multiple of reference MIC (MICref) and timepoint.

ASM does not own the copyrights to Supplemental Material that may be linked to, or accessed through, an article. The authors have granted ASM a non-exclusive, world-wide license to publish the Supplemental Material files. Please contact the corresponding author directly for reuse.
