# Supplementary material for: SMT-738: a novel small-molecule inhibitor of bacterial lipoprotein transport targeting Enterobacteriaceae
Source: Antimicrob Agents Chemother. 2023 Dec 12;68(1):e00695-23. doi: 10.1128/aac.00695-23 (PMC10777851; doi:10.1128/aac.00695-23)
Supplement: Supplementary Fig. S1 — Clustal Omega alignment of LolC (A) and LolE (B) showing structural features and mutated residues. [file aac.00695-23-s0001.ppt]

## Slide 1
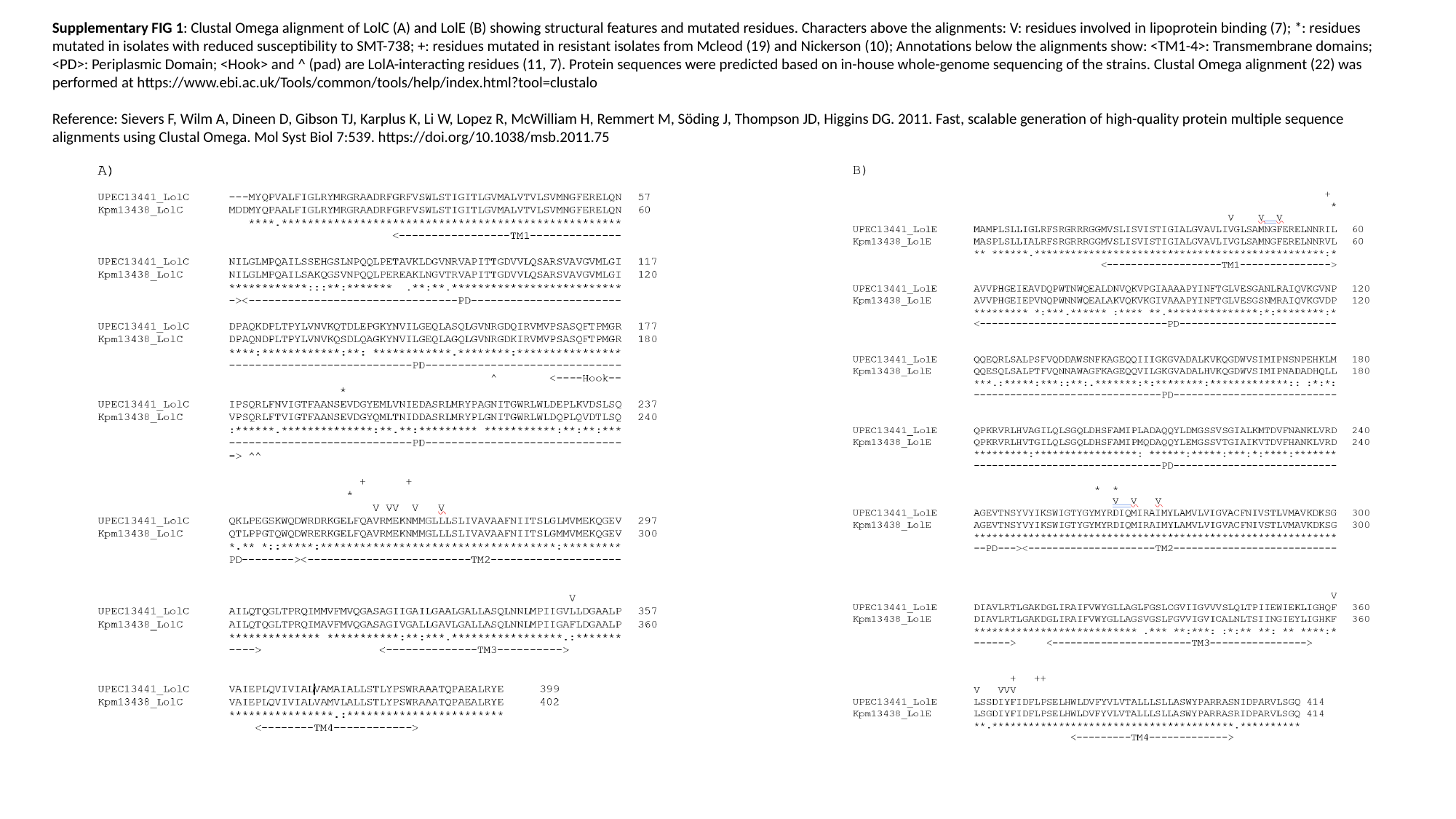

Supplementary FIG 1: Clustal Omega alignment of LolC (A) and LolE (B) showing structural features and mutated residues. Characters above the alignments: V: residues involved in lipoprotein binding (7); *: residues mutated in isolates with reduced susceptibility to SMT-738; +: residues mutated in resistant isolates from Mcleod (19) and Nickerson (10); Annotations below the alignments show: <TM1-4>: Transmembrane domains; <PD>: Periplasmic Domain; <Hook> and ^ (pad) are LolA-interacting residues (11, 7). Protein sequences were predicted based on in-house whole-genome sequencing of the strains. Clustal Omega alignment (22) was performed at https://www.ebi.ac.uk/Tools/common/tools/help/index.html?tool=clustalo
Reference: Sievers F, Wilm A, Dineen D, Gibson TJ, Karplus K, Li W, Lopez R, McWilliam H, Remmert M, Söding J, Thompson JD, Higgins DG. 2011. Fast, scalable generation of high-quality protein multiple sequence alignments using Clustal Omega. Mol Syst Biol 7:539. https://doi.org/10.1038/msb.2011.75
