# Supplementary material for: SMT-738: a novel small-molecule inhibitor of bacterial lipoprotein transport targeting Enterobacteriaceae
Source: Antimicrob Agents Chemother. 2023 Dec 12;68(1):e00695-23. doi: 10.1128/aac.00695-23 (PMC10777851; doi:10.1128/aac.00695-23)
Supplement: Supplementary Fig. S2 — Artemis plot showing the location of mini-transposon insertions around lpp (A) and nlpE(cutF) (B) in E. coli NCTC 13441 libraries treated with SMT-738. [file aac.00695-23-s0002.ppt]

## Slide 1
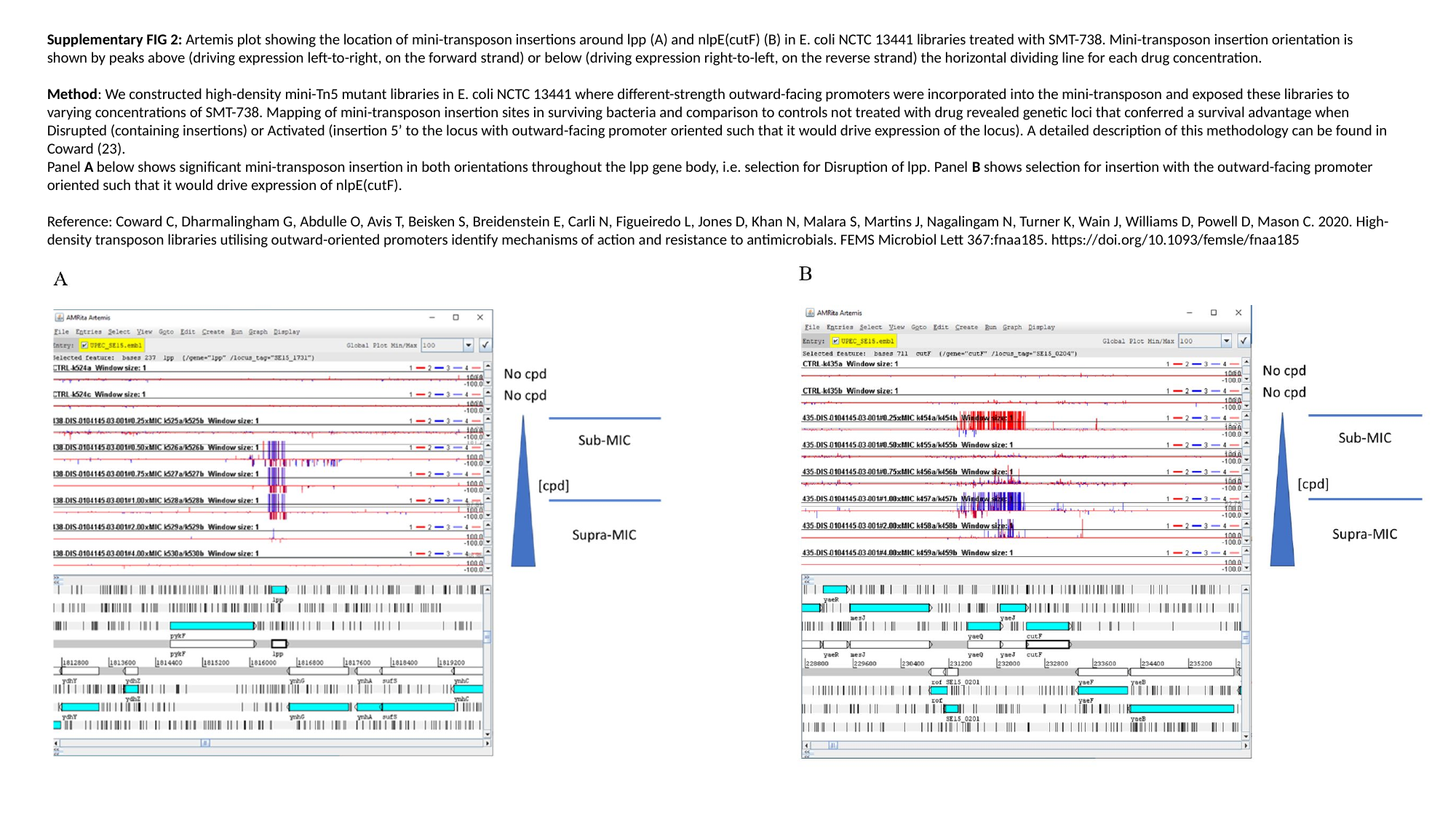

Supplementary FIG 2: Artemis plot showing the location of mini-transposon insertions around lpp (A) and nlpE(cutF) (B) in E. coli NCTC 13441 libraries treated with SMT-738. Mini-transposon insertion orientation is shown by peaks above (driving expression left-to-right, on the forward strand) or below (driving expression right-to-left, on the reverse strand) the horizontal dividing line for each drug concentration.
Method: We constructed high-density mini-Tn5 mutant libraries in E. coli NCTC 13441 where different-strength outward-facing promoters were incorporated into the mini-transposon and exposed these libraries to varying concentrations of SMT-738. Mapping of mini-transposon insertion sites in surviving bacteria and comparison to controls not treated with drug revealed genetic loci that conferred a survival advantage when Disrupted (containing insertions) or Activated (insertion 5’ to the locus with outward-facing promoter oriented such that it would drive expression of the locus). A detailed description of this methodology can be found in Coward (23).
Panel A below shows significant mini-transposon insertion in both orientations throughout the lpp gene body, i.e. selection for Disruption of lpp. Panel B shows selection for insertion with the outward-facing promoter oriented such that it would drive expression of nlpE(cutF).
Reference: Coward C, Dharmalingham G, Abdulle O, Avis T, Beisken S, Breidenstein E, Carli N, Figueiredo L, Jones D, Khan N, Malara S, Martins J, Nagalingam N, Turner K, Wain J, Williams D, Powell D, Mason C. 2020. High-density transposon libraries utilising outward-oriented promoters identify mechanisms of action and resistance to antimicrobials. FEMS Microbiol Lett 367:fnaa185. https://doi.org/10.1093/femsle/fnaa185
