# Supplementary material for: SMT-738: a novel small-molecule inhibitor of bacterial lipoprotein transport targeting Enterobacteriaceae
Source: Antimicrob Agents Chemother. 2023 Dec 12;68(1):e00695-23. doi: 10.1128/aac.00695-23 (PMC10777851; doi:10.1128/aac.00695-23)
Supplement: Supplementary Fig. S3 — SMT-738 concentration-time profiles in plasma after IV infusion administration. [file aac.00695-23-s0003.ppt]

## Slide 1
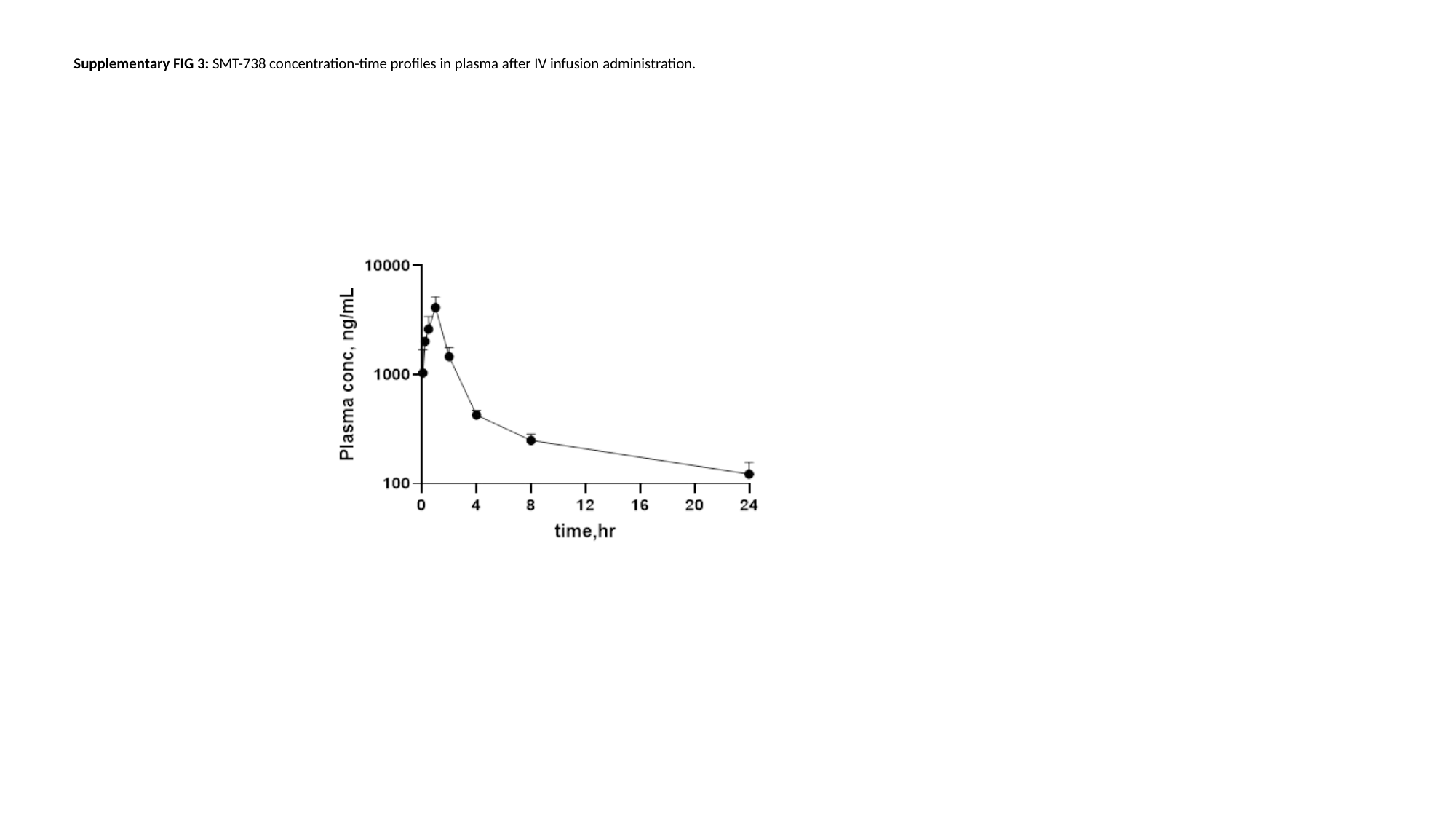

Supplementary FIG 3: SMT-738 concentration-time profiles in plasma after IV infusion administration.
