# Supplementary material for: SMT-738: a novel small-molecule inhibitor of bacterial lipoprotein transport targeting Enterobacteriaceae
Source: Antimicrob Agents Chemother. 2023 Dec 12;68(1):e00695-23. doi: 10.1128/aac.00695-23 (PMC10777851; doi:10.1128/aac.00695-23)
Supplement: Supplementary Table S1 — Isolates used in Table 2. [file aac.00695-23-s0004.pdf]

**Supplementary Table 1**: Isolates used in Table 2. Origin as well as molecular background of the isolates are shown

| Country | OrganismName | OrganismFamilyName | YearCollected | BodyLocation | Molecular Summary |
|---|---|---|---|---|---|
| India | *Escherichia coli* | Enterobacteriaceae | 2018 | GU: Urine | TEM-OSBL;NDM-5;CTX-M-15;CMY-42 |
| India | *Escherichia coli* | Enterobacteriaceae | 2018 | GU: Urine | TEM-OSBL;OXA-181;NDM-5;CMY-42 |
| India | *Escherichia coli* | Enterobacteriaceae | 2018 | Respiratory: Endotracheal aspirate | NDM-5;CMY-2-TYPE |
| India | *Escherichia coli* | Enterobacteriaceae | 2018 | GU: Urine | CTX-M-15;NDM-5 |
| India | *Escherichia coli* | Enterobacteriaceae | 2018 | Respiratory: Endotracheal aspirate | TEM-OSBL;NDM-5;CTX-M-15 |
| India | *Escherichia coli* | Enterobacteriaceae | 2018 | Respiratory: Bronchoalveolar lavage | TEM-OSBL;NDM-4;CTX-M-15 |
| India | *Escherichia coli* | Enterobacteriaceae | 2018 | Respiratory: Endotracheal aspirate | TEM-OSBL;NDM-5;CTX-M-15;CMY-42 |
| India | *Escherichia coli* | Enterobacteriaceae | 2018 | Respiratory: Bronchoalveolar lavage | TEM-OSBL;NDM-5;CTX-M-15;CMY-42 |
| India | *Escherichia coli* | Enterobacteriaceae | 2018 | Respiratory: Endotracheal aspirate | DHA;TEM-OSBL;NDM-5;CMY-2-TYPE |
| India | *Escherichia coli* | Enterobacteriaceae | 2018 | CVS: Blood | TEM-OSBL;OXA-484;NDM-5 |
| India | *Escherichia coli* | Enterobacteriaceae | 2018 | CVS: Blood | NDM-5;CTX-M-15 |
| India | *Escherichia coli* | Enterobacteriaceae | 2018 | Respiratory: Sputum | TEM-OSBL;OXA-181;NDM-5;CMY-2-TYPE |
| India | *Escherichia coli* | Enterobacteriaceae | 2018 | Respiratory: Sputum | NDM-5;CTX-M-15 |
| India | *Escherichia coli* | Enterobacteriaceae | 2018 | GU: Urine | NDM-5;CTX-M-15;TEM-OSBL |
| India | *Escherichia coli* | Enterobacteriaceae | 2018 | GU: Urine | CTX-M-15;CMY-2;TEM-OSBL;NDM-5 |
| India | *Klebsiella pneumoniae* | Enterobacteriaceae | 2018 | Respiratory: Endotracheal aspirate | TEM-OSBL;SHV-OSBL;OXA-232;NDM-1;CTX-M-15 |
| India | *Klebsiella pneumoniae* | Enterobacteriaceae | 2018 | CVS: Blood | TEM-OSBL;SHV-OSBL;OXA-232;NDM-1;CTX-M-15 |
| India | *Klebsiella pneumoniae* | Enterobacteriaceae | 2018 | CVS: Blood | TEM-OSBL;SHV-OSBL;NDM-5;CTX-M-15 |
| India | *Klebsiella pneumoniae* | Enterobacteriaceae | 2018 | CVS: Blood | DHA;SHV-OSBL;NDM-4;CTX-M-15 |
| India | *Klebsiella pneumoniae* | Enterobacteriaceae | 2018 | GU: Urine | NDM-5;CTX-M-15;SHV-OSBL;TEM-OSBL |
| India | *Klebsiella pneumoniae* | Enterobacteriaceae | 2018 | GU: Urine | TEM-OSBL;SHV-OSBL;NDM-5 |
| India | *Klebsiella pneumoniae* | Enterobacteriaceae | 2018 | CVS: Blood | OXA-232;NDM-5;CTX-M-15;SHV-OSBL;TEM-OSBL |
| India | *Klebsiella pneumoniae* | Enterobacteriaceae | 2018 | GU: Urine | TEM-OSBL;SHV-OSBL;OXA-181;NDM-5;CTX-M-15 |
| India | *Klebsiella pneumoniae* | Enterobacteriaceae | 2018 | Respiratory: Sputum | SHV-OSBL;OXA-181;NDM-5;CTX-M-15 |
| India | *Klebsiella pneumoniae* | Enterobacteriaceae | 2018 | Respiratory: Bronchoalveolar lavage | TEM-OSBL;SHV-OSBL;OXA-232;NDM-5;CTX-M-15 |
| India | *Klebsiella pneumoniae* | Enterobacteriaceae | 2018 | GU: Urine | TEM-OSBL;SHV-OSBL;OXA-181;NDM-5;CTX-M-15 |
| India | *Klebsiella pneumoniae* | Enterobacteriaceae | 2018 | Respiratory: Lungs | TEM-OSBL;SHV-OSBL;OXA-181;NDM-5;CTX-M-15 |
| India | *Klebsiella pneumoniae* | Enterobacteriaceae | 2018 | Respiratory: Sputum | TEM-OSBL;SHV-OSBL;CTX-M-15;NDM-5;OXA-48 |
| India | *Klebsiella pneumoniae* | Enterobacteriaceae | 2018 | GU: Urine | TEM-OSBL;OXA-232;CTX-M-15;SHV-OSBL |
| India | *Klebsiella pneumoniae* | Enterobacteriaceae | 2018 | CVS: Blood | SHV-OSBL;NDM-5;CTX-M-15 |
