# Supplementary material for: SMT-738: a novel small-molecule inhibitor of bacterial lipoprotein transport targeting Enterobacteriaceae
Source: Antimicrob Agents Chemother. 2023 Dec 12;68(1):e00695-23. doi: 10.1128/aac.00695-23 (PMC10777851; doi:10.1128/aac.00695-23)
Supplement: Supplementary Table S2 — Minimum inhibitory concentration (MIC μg/mL) against 100 clinical bacterial species. [file aac.00695-23-s0005.pdf]

Supplementary Table 2: Minimum Inhibitory Concentration (MIC µg/mL) against 100 clinical bacterial species representing Gram-negative, Gram-positive and aneraerobic pathogens. Bacteria belonging to the Enterobacterales family are highlighted in grey

Organism	Body Location	SMT-738	Organism	Body Location	SMT-738
Acinetobacter baumannii	GU: Urine	>64	*Lactobacillus paracasei*	bloodstream infection	>128
Acinetobacter calcoaceticus	Respiratory: Endotracheal aspirate	>64	*Lactobacillus plantarum*	bloodstream infection	>128
Actinomyces neuii	anaerobes	>128	*Lactobacillus rhamnosus*	bloodstream infection	>128
Actinomyces odontolyticus	anaerobes	>128	*Lactococcus garvieae*	bloodstream infection	>128
Actinomyces oris	anaerobes	>128	*Lactococcus lactis*	bloodstream infection	>128
Actinomyces oris	anaerobes	>128	*Moraxella catarrhalis*	Respiratory: Bronchoalveolar lavage	>64
Bacteroides caccae	anaerobes	>128	*Morganella morganii*	GU: Urine	>64
Bacteroides caccae	anaerobes	>128	*Morganella morganii*	Respiratory: Sputum	64
Bacteroides fragilis	anaerobes	>64	*Parabacteroides distasonis*	anaerobes	>64
Bacteroides ovatus	anaerobes	>64	*Parabacteroides distasonis*	anaerobes	>128
Bacteroides thetaiotaomicron	anaerobes	>64	*Parabacteroides goldsteinii*	anaerobes	>64
Bacteroides thetaiotaomicron	anaerobes	>128	*Parabacteroides johnsonii*	anaerobes	>64
Bacteroides uniformis	anaerobes	>128	*Parabacteroides merdae*	anaerobes	>64
Bacteroides uniformis	anaerobes	>128	*Parvimonas micra*	anaerobes	>64
Bifidobacterium adolescentis	anaerobes	>128	*Pasteurella multocida*	Respiratory: Endotracheal aspirate	4
Bifidobacterium bifidum	anaerobes	>128	*eptostreptococcus anaerobius*	anaerobes	>64
Bifidobacterium breve	anaerobes	>128	*Peptostreptococcus magnus*	anaerobes	>64
Bifidobacterium longum	anaerobes	>128	*Plesiomonas shigelloides*	CVS: Blood	0.12
Burkholderia cepacia	CVS: Blood	>64	*Prevotella bivia*	anaerobes	>64
Burkholderia multivorans	Respiratory: Sputum	>64	*Prevotella melaninogenica*	anaerobes	>64
Campylobacter jejuni	bloodstream infection	>128	*Proteus hauseri*	GU: Urine	8
Cedecea, non-speciated	GU: Urine	0.5	*Proteus mirabilis*	Bodily Fluids: Abscess / Pus	16
Citrobacter freundii	CVS: Blood	0.5	*Proteus penneri*	Bodily Fluids: Peritoneal	8
Citrobacter koseri	GU: Kidneys	0.5	*Proteus vulgaris*	GU: Urinary Bladder	32
Closridium difficile	anaerobes	>64	*Providencia alcalifaciens*	GU: Urine	8
Closridium perfringens	anaerobes	>64	*Providencia rettgeri*	CVS: Blood	2
Clostridium ramosum	anaerobes	>128	*Providencia stuartii*	CVS: Blood	2
Clostridium sordellii	anaerobes	>128	*Pseudomonas aeruginosa*	GU: Urine	>64
Clostridium tertium	anaerobes	>128	*Pseudomonas putida*	GU: Urine	>64
Corynebacterium amycolatum	bloodstream infection	>128	*Ralstonia non-speciated*	Respiratory: Bronchoalveolar lavage	>64
Corynebacterium nuruki	bloodstream infection	>128	*Ralstonia pickettii*	GI: Rectum	>64
ynebacterium pseudodiptheritic	bloodstream infection	>128	*Raoultella ornithinolytica*	Respiratory: Endotracheal aspirate	0.25
Corynebacterium striatum	bloodstream infection	>128	*Raoultella planticola*	Respiratory: Other	0.25
Cutibacterium acnes	anaerobes	>128	*Salmonella, non-speciated*	CVS: Blood	0.5
Edwardsiella tarda	Bodily Fluids: Abscess / Pus	32	*Serratia liquefaciens*	Respiratory: Sputum	0.5
Eggerthella lenta	anaerobes	>128	*Serratia marcescens*	GU: Urine	1
Enterobacter cloacae	CVS: Blood	0.5	*Serratia odorifera*	CVS: Blood	0.5
Enterococcus faecalis	CVS: Blood	>64	*Serratia rubidaea*	Respiratory: Other	2
Enterococcus faecium	Respiratory: Endotracheal aspirate	>64	*Shigella boydii*	GU: Urine	0.5
Erwinia, non-speciated	Bodily Fluids: Peritoneal	1	*Shigella flexneri*	GU: Urine	0.5
Escherichia coli	GU: Urine	0.5	*Shigella sonnei (Group D)*	GastroIntestinal (GI)	0.12
Finegoldia magna	anaerobes	>64	*Staphylococcus aureus*	Respiratory: Sputum	>64
Fusobacterium necrophorum	anaerobes	>128	*Staphylococcus epidermidis*	Respiratory: Endotracheal aspirate	>64
Fusobacterium nucleatum	anaerobes	>128	*Streptococcus agalactiae*	Respiratory: Endotracheal aspirate	>64
Haemophilus influenzae	Respiratory: Bronchoalveolar lavage	1	*Streptococcus parasanguinis*	Respiratory: Sputum	>64
Haemophilus parainfluenzae	Respiratory: Sputum	1	*Streptococcus pneumoniae*	N/A	>128
Hafnia alvei	Bodily Fluids: Abscess / Pus	32	*Streptococcus salivarius*	Bodily Fluids: Abscess / Pus	>64
Helicobacter pylori	other site	>128	*Unspeciated Eubacterium*	anaerobes	>128
Klebsiella pneumoniae	CVS: Blood	0.5	*Veillonella parvula*	anaerobes	>128
Kluyvera ascorbata	Respiratory: Sputum	1	*Yersinia enterocolitica*	Respiratory: Sputum	0.5
			Yersinia pseudotuberculosis	GI: Liver	1
