# Supplementary material for: SMT-738: a novel small-molecule inhibitor of bacterial lipoprotein transport targeting Enterobacteriaceae
Source: Antimicrob Agents Chemother. 2023 Dec 12;68(1):e00695-23. doi: 10.1128/aac.00695-23 (PMC10777851; doi:10.1128/aac.00695-23)
Supplement: Supplementary Table S3 — Frequency of resistance (or limit of detection) for SMT-738 at the indicated multiple of reference MIC (MICref) and timepoint. [file aac.00695-23-s0006.pdf]

Supplementary Table 3: Frequency of resistance (or limit of detection) for SMT-738 at the indicated multiple of reference MIC (MICref) and timepoint (A: 24 h; B: 48h). 0.78 µM (= 0.3 µg/mL) and 3.13 µM (= 1.22 µg/mL). [1]Number of experiments in which colonies were observed; [2]Frequency of resistance range from experiments with colonies observed; [3]Number of experiments in which no colonies were observed; [4]Limit of detection range from experiments with no colonies observed

**A)**				**24 h**			
**Cpd**	**Strain**	**MICref (µM)**	**xMICref**	**FoR Exps[1]**	**FoR Range[2]**	**LoD Exps[3]**	**LoD Range[4]**
SMT-738	UPEC13441	0.781	4	3	2.40E-10 to 1.20E-09	2	<9.76E-10 to <3.00E-09
			8	1	7.20E-10	4	<5.07E-10 to <2.44E-09
	Kpm13438	3.13	4	2	1.13E-09 to 1.43E-09	1	<2.26E-09
			8	2	3.75E-10 to 4.76E-10	1	<2.26E-09

**B)**				**48 h**			
**Cpd**	**Strain**	**MICref (µM)**	**xMICref**	**FoR Exps[1]**	**FoR Range[2]**	**LoD Exps[3]**	**LoD Range[4]**
SMT-738	UPEC13441	0.781	4	1	1.52E-09	2	<9.76E-10 to <3.00E-09
			8	None		3	<5.07E-10 to <2.44E-09
	Kpm13438	3.13	4	1	1.50E-09	1	<2.26E-09
			8	1	3.75E-10	1	<2.26E-09
